# Definitive Surgical Management of Refractory Amiodarone-Induced Thyrotoxicosis in a Cardiac Patient: A Case Report

**DOI:** 10.7759/cureus.83354

**Published:** 2025-05-02

**Authors:** Islam Shah, Jonathan Fabricante, Fahas Ali Vattiyam Veettil

**Affiliations:** 1 Internal Medicine (General Medicine), District Headquarter Hospital, Daggar, PAK; 2 Internal Medicine, Khyber Teaching Hospital, Peshawar, PAK; 3 Medicine, Khyber Medical College, Peshawar, PAK; 4 Diabetes and Endocrinology, The Princess Alexandra Hospital NHS Trust, Harlow, GBR; 5 Medicine, The Princess Alexandra Hospital NHS Trust, Harlow, GBR

**Keywords:** ait, amiodarone-induced thyrotoxicosis, atrial fibrillation, endocrine surgery, refractory thyrotoxicosis, thyroidectomy

## Abstract

Amiodarone-induced thyrotoxicosis (AIT) presents a significant therapeutic challenge, particularly in patients with underlying cardiac comorbidities. We present a 58-year-old man with a background history of ischemic heart disease and atrial fibrillation who developed AIT following long-term amiodarone use. Despite the escalation of medical therapy, he remained clinically and biochemically thyrotoxic with symptomatic atrial fibrillation. Definitive management via total thyroidectomy was performed, resulting in clinical stabilization. This case highlights the importance of early multidisciplinary input and the role of total thyroidectomy in managing refractory AIT in high-risk cardiac patients.

## Introduction

Amiodarone, a class III antiarrhythmic agent, is well known for its association with thyroid dysfunction due to its high iodine content and direct cytotoxic effects on thyroid tissue [1]. Amiodarone-induced thyrotoxicosis (AIT) is broadly classified into two types: type 1 AIT, which results from iodine-induced hormone synthesis in patients with underlying thyroid disease, and type 2 AIT, destructive thyroiditis seen in previously normal glands, caused by direct follicular cell toxicity and release of preformed hormone [2,3].

In type 2 AIT, high tissue concentrations of the drug and its metabolites lead to the inflammatory disruption of thyroid follicles and unregulated thyroid hormone release. This subtype can be particularly challenging to manage in patients with cardiovascular comorbidities, as the resulting thyrotoxicosis may precipitate life-threatening arrhythmias [2]. While medical therapy-including thionamides and glucocorticoids-is first-line, refractory cases often require surgical intervention. Surgical management of AIT has been well documented in previous literature, including Mayo Clinic experience and European Thyroid Association guidelines, supporting thyroidectomy as a definitive option in severe or unresponsive cases [4,5]. In this report, we describe a patient with type 2 AIT unresponsive to maximal medical therapy who achieved rapid resolution following total thyroidectomy.

## Case presentation

A 58-year-old male patient with a history of hypertension, ischemic heart disease, asthma, benign prostatic hyperplasia, and persistent atrial fibrillation (AF) was referred for endocrine assessment following a diagnosis of AIT. He had been treated with amiodarone 200 mg per day for two years, which was discontinued after catheter ablation for AF in February 2024. Approximately four months after discontinuing amiodarone, the patient initially presented to the emergency department after collapsing at work. He described palpitations, tremors, and malaise. Biochemical evaluation confirmed thyrotoxicosis, and he was commenced on carbimazole 15 mg twice a day (Table 1).

**Table 1:** Initial thyroid function test on presentation TSH: thyroid-stimulating hormone

Test	Initial presentation	Reference range		
TSH	0.01 mIU/L	0.4–4.0 mIU/L		
Free T4	53.2 pmol/L	9.0–25.0 pmol/L		
Free T3	15.4 pmol/L	3.1–6.8 pmol/L		

Due to poor clinical response, the carbimazole dose was increased to 20 mg twice daily by his general practitioner. However, after four weeks, he re-presented with worsening symptoms including AF with fast ventricular response (FVR), tremors, and restlessness.

Due to worsening clinical status and suboptimal response to initial therapy, medical management was escalated to include propylthiouracil (PTU) 200 mg three times daily, intravenous hydrocortisone (later transitioned to oral prednisolone 40 mg), and cholestyramine 8 mg three times daily. Rate control was attempted with bisoprolol 10 mg, but this was insufficient alone; therefore, digoxin was added with a loading dose followed by a maintenance dose of 250 mcg daily, as per cardiology advice. In light of the severity of symptoms and limited biochemical improvement, the Burch-Wartofsky Point Scale (BWPS) was applied, with an estimated score of 40, indicating impending thyroid storm and supporting the need for intensified medical treatment.

His laboratory results and ultrasound findings were consistent with type 2 AIT, as shown in Table 2.

**Table 2:** The result of thyroid function test and thyroid antibody test TSH: thyroid-stimulating hormone; TPO: thyroid peroxidase

Test	Initial result (at 4th week)	Follow-up result (at 8th week)	Reference range
Free T4	44.8 pmol/L	26.3 pmol/L	9.0–25.0 pmol/L
Free T3	19.9 pmol/L	5.6 pmol/L	3.1–6.8 pmol/L
TSH	<0.01 mIU/L	<0.01 mIU/L	0.4–4.0 mIU/L
TPO antibodies	<9.0 IU/mL	<9.0 IU/mL	0–34 IU/mL
TSH receptor antibodies	<0.80 IU/L	<0.80 IU/L	<1.75 IU/L

Thyroid ultrasound revealed a mildly heterogeneous gland with no discrete nodules or increased vascularity-features consistent with type 2 AIT. Due to persistent symptoms and only partial biochemical response, the case was referred to endocrine surgery. A total thyroidectomy was planned. Preoperative optimization included PTU, prednisolone, cholestyramine, digoxin, bisoprolol, and Lugol’s iodine solution for 10 days. The patient underwent total thyroidectomy without complication.

Postoperatively, he was monitored in the high-dependency unit and transferred to the ward. Prednisolone was tapered, and levothyroxine replacement therapy was initiated. He was discharged in stable condition with the resolution of tremors and improved heart rate control.

The patient was followed up in the Same Day Emergency Care (SDEC) unit at two and four weeks post-discharge. He showed continued clinical and biochemical improvement, achieving euthyroid status and restoration of normal sinus rhythm with a well-controlled heart rate (Table 3).

**Table 3:** Thyroid function test at two and four weeks post-discharge in SDEC TSH: thyroid-stimulating hormone; SDEC: Same Day Emergency Care

Test	2 weeks	4 weeks
TSH (mIU/L)	0.01	0.48
Free T4 (pmol/L)	15.1	12.5
Free T3 (pmol/L)	4.9	3.7

## Discussion

AIT presents a unique clinical challenge, especially in individuals with preexisting cardiac conditions. AIT is typically categorized into two main types: type 1 AIT, which results from iodine-induced excess hormone synthesis in patients with underlying thyroid pathology, and type 2 AIT, destructive thyroiditis causing the release of preformed thyroid hormone in those with previously normal thyroid glands [1]. Mixed forms are also recognized, and distinguishing between them can be difficult but is important as it affects the management strategy.

Our patient presented with clinical features and imaging findings suggestive of type 2 AIT. This subtype often responds to glucocorticoids, yet in severe or refractory cases, as seen here, a more aggressive approach may be required. Medical therapy typically includes thionamides, corticosteroids, beta-blockers, and sometimes adjunctive agents such as cholestyramine and potassium perchlorate [2]. However, medical therapy may not always be sufficient or rapid enough to control thyrotoxicosis in patients with unstable cardiac conditions, where delayed control may result in significant morbidity or mortality [3].

Surgical intervention in the form of total thyroidectomy offers definitive treatment. Although surgery carries inherent risks, particularly in hyperthyroid and cardiac patients, it has shown favorable outcomes when performed in experienced centers after preoperative optimization. Recent literature supports early consideration of thyroidectomy in patients who are critically ill, refractory to treatment, or at risk of cardiac decompensation [4,5]. A retrospective study by Bogazzi et al. and subsequent guideline reviews by the European Thyroid Association and the American Thyroid Association support thyroidectomy as a safe and effective intervention in cases of refractory AIT [1,4].

In our case, despite escalated multimodal medical therapy, the patient continued to experience symptoms and biochemical evidence of thyrotoxicosis, with AF and a high heart rate. Surgery was well tolerated following appropriate preoperative preparation, including Lugol's iodine to reduce gland vascularity. This highlights the critical role of a multidisciplinary team-including endocrinologists, cardiologists, and endocrine surgeons-in ensuring a successful outcome.

## Conclusions

This case highlights the complexity of managing AIT, particularly in cardiac patients unresponsive to medical therapy. Type 2 AIT can be challenging to treat due to its inflammatory nature and the potential for life-threatening complications such as thyroid storm. In our case, despite the escalation of antithyroid and supportive therapy, only surgical intervention achieved a definitive resolution. Early recognition, appropriate subclassification, and a multidisciplinary approach are essential in managing refractory AIT, with total thyroidectomy remaining a viable and life-saving option when medical therapy fails.
